# Assessing thickness and stiffness of superficial/deep masticatory muscles in orofacial pain: an ultrasound and shear wave elastography study

**DOI:** 10.1080/07853890.2023.2261116

**Published:** 2023-10-04

**Authors:** Yunn-Jy Chen, Hui-Yi Lin, Cheng-An Chu, Wei-Ting Wu, Lan-Rong Chen, Levent Özçakar, Ke-Vin Chang

**Affiliations:** a Department of Dentistry, National Taiwan University Hospital, Taipei, Taiwan; b Department of Physical Medicine and Rehabilitation and Community and Geriatric Research Center, National Taiwan University Hospital, Taipei, Taiwan; c Department of Physical Medicine and Rehabilitation, College of Medicine, National Taiwan University Hospital, National Taiwan University, Taipei, Taiwan; d Department of Physical and Rehabilitation Medicine, Hacettepe University Medical School, Ankara, Turkey; e Center for Regional Anesthesia and Pain Medicine, Wang-Fang Hospital, Taipei Medical University, Taipei, Taiwan

**Keywords:** Sonography, sonoelastography, orofacial pain, temporo-mandibular joint, mastication

## Abstract

**Introduction:**

Sonoelastography has been increasingly used for non-invasive evaluation of the mechanical features of human tissues. The interplay between orofacial pain and regional muscle activity appears clinically paramount, although only few imaging studies have investigated this association. Using shear wave sonoelastography (SWS), this study ascertained whether orofacial pain induced alterations in the stiffness of superficial and deep masticatory muscles.

**Methods:**

All participants were systematically evaluated for oral/facial-related conditions, including the area and intensity of pain. SWS was applied to measure the stiffness of the bilateral masseter, temporalis, and lateral pterygoid muscles. The association between orofacial pain and muscle stiffness/thickness was investigated using a generalized estimating equation for adjusting the influence of age, sex, laterality, and body mass index on muscle thickness/stiffness.

**Results:**

A total of 98 participants were included in the present study: 48 asymptomatic controls, 13 patients with unilateral pain, and 37 patients with bilateral orofacial pain. The reliability, quantified by the intraclass correlation coefficient for muscle stiffness measurement, ranged from 0.745 to 0.893. Orofacial pain at the individual muscle level was significantly associated with masseter muscle stiffness. A trend of increased stiffness (*p* = 0.06) was also observed in relation to the painful side of the temporalis muscle. No significant correlation was identified between the numeric rating scales for pain and stiffness measurements.

**Conclusions:**

SWS provides reliable stiffness measurements for the superficial and deep masticatory muscles. The ipsilateral masseter and temporalis muscles might be stiffer than those on the side without orofacial pain. Future studies using the present sonoelasotography protocol can be designed to investigate the stiffness changes in the target muscles after interventions.

## KEY MESSAGES

- Shear wave sonoelastography (SWS) can reliably assess the stiffness of masticatory muscles.
- Orofacial pain, particularly affecting the ipsilateral masseter muscles, exhibited increased stiffness, with a similar trend observed in the temporalis muscle as revealed by SWS. However, the stiffness of the lateral pterygoid muscle appeared to remain unaffected.
- These findings establish a foundational framework for the objective and quantitative assessment of orofacial pain and indicate the potential utility of SWS as a tool for evaluating treatment outcomes.

## Introduction

Orofacial pain, defined as 'pain perceived in the face and/or oral cavity' by the International Association for the Study of Pain (IASP), has been reported to have a prevalence between 7% and 16% [1]. Women are at a higher risk of orofacial pain than men. Furthermore, bruxism, daytime clenching, unilateral chewing, and heavy lifting at work have been related to orofacial pain [2]. The clinical scenario can be derived from a variety of causes, such as temporo-mandibular joint disorders, burning mouth syndrome, atypical odontalgia, and trigeminal neuralgia [3]. The underlying pathophysiology is complex and multifactorial, encompassing decreased dopamine secretion in the putamen, intraoral somatosensory dysfunction, and small fiber sensory trigeminal neuropathy [4]. Several muscles are commonly involved in the development of orofacial pain, including the masseter, buccinator, temporalis, and lateral pterygoid. Orofacial pain can originate from muscle hyperactivity [5]. Further, pain in the orofacial region could limit jaw muscle movement as a protective mechanism to prevent overuse injury [5]. Although the interplay between orofacial pain and regional muscles appears clinically paramount, there are only few imaging studies on their association.

Ultrasound has emerged as a reliable and cost-effective tool for evaluating muscle quality and quantity [6]. The absence or disruption of the fibrillary arrangement and presence of a visible gap can be identified on B-mode imaging after various grades of injury [7]. With regard to disused muscles, increased echogenicity and decreased thickness secondary to fatty infiltration and atrophy are sonographic hallmarks [8]. Using a standardized scanning protocol, satisfactory reliability can be achieved for thickness measurements of the masseter, temporalis, and lateral pterygoid muscles [9].

Recently, an emerging technique, sonoelastography, has been increasingly used for noninvasive evaluation of the mechanical features of human tissues, such as rotator cuff tendons [10], median nerves [11] and tongue muscles [12]. A recent systematic review, including 16 clinical studies, highlighted the potential of grading the severity of temporo-mandibular joint disorders using sonoelastography [13]. However, most of the existing studies focused on the elasticity of the masseter muscles and recruited participants with or without temporo-mandibular joint disorders. There is a scarcity of evidence regarding stiffness changes in other masticatory muscles among patients with orofacial pain, even though such changes could potentially serve as predictors for the success of orofacial pain treatment. This underscores the urgent need for further research in this area. Therefore, the present study aimed to investigate the association of orofacial pain with the stiffness of superficial/deep masticatory muscles using shear wave sonoelastography (SWS). We proposed that orofacial pain might result in heightened tension within the masticatory muscles, potentially leading to increased stiffness as assessed through SWS.

## Methods

### Ethics statement

The research protocol was approved by the Institutional Review Board of the National Taiwan University Hospital (201912016RIN), and all eligible participants provided written informed consent before enrollment. The study was conducted in accordance with relevant guidelines and regulations (Declaration of Helsinki).

### Participants

Participants for this study were recruited from the Department of Dentistry at the National Taiwan University Hospital, as well as its Bei-Hu branch. Inclusion criteria encompassed individuals who were: (1) aged over 20 years, (2) possessed intact cognitive abilities enabling them to respond to inquiries effectively, and (3) free from uncontrolled medical conditions, such as unstable angina.

On the other hand, exclusion criteria included those with: (1) a history of previous facial trauma, (2) dental treatment within the preceding six months, (3) a medical history of trigeminal neuralgia or neuromuscular diseases like myasthenia gravis and myositis, and (4) active facial or neck infection.

### Clinical evaluation

All participants were evaluated systematically for oral/facial-related conditions. First, the site (e.g. temporo-mandibular joint, temporal fossa, infra-zygomatic region, and mandibular ramus) and intensity of orofacial pain and tenderness were documented using a numeric rating scale (0-10). Regarding the position of the incisors, the presence of horizontal incisal overjet, vertical incisal overlap, and midline incisal deviation was recorded. The trajectory (straight, corrected deviation, and uncorrected deviation), width, and presence of clicking/locking of mouth opening were noted.

### Ultrasound assessment

Images were obtained using an ultrasound system (Aplio 300 Platinum platform, Toshiba, Tokyo, Japan) along with convex (PVT-375SC, 50 mm wide, 1.5-6 MHz) and linear (PLT-1005BT, 58 mm wide, 3.8-10 MHz) transducers. While using the linear transducer, the scanning depth was set at 3 cm with the focus fixed at 1.5 cm; whereas the scanning depth was switched to 6 cm with the focus changed to 4.5 cm while using the convex transducer. The targets for thickness and stiffness measurements were the masseter, temporalis, and lateral pterygoid muscles. During the assessment of the former two muscles, the participants were asked to lightly close their mouth with approximation of the upper and lower lips. They were required to open their mouth maximally for clear visualization of the lateral pterygoid muscle. During the examination of the masseter and temporalis muscles, we encouraged patients to remain in a relaxed state. However, it was necessary to induce a slight increase in muscle tension when examining the lateral pterygoid muscle, as maximal mouth opening was required for complete visualization. When participants were asked to open their mouths, they did so voluntarily. We did not employ any oral appliances to keep the mouth open.

**Figure 1.**
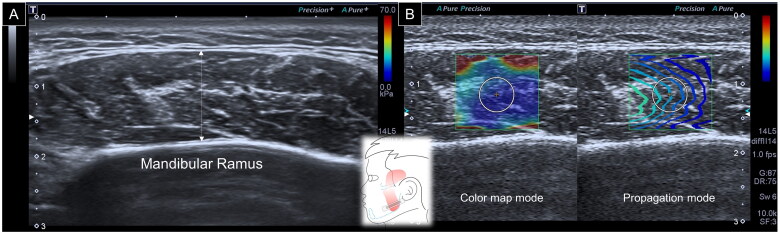
Ultrasound imaging for thickness **(A)** and stiffness **(B)** measurements of the masseter muscle. White dashed line, maximal muscle thickness.

To assess the masseter muscle, a linear transducer was placed in the horizontal plane at the midpoint of the mandibular ramus. The posterior portion of the muscle was visualized in the mandibular cortex. The maximal vertical distance from the superficial muscle fascia to the periosteum surface was defined as its thickness [9]. Later, the SWS mode was turned on with the color elastogram on the left-half screen and the propagation mode on the right-half screen. An elastogram was used to ensure that there were no color-filling defects inside the region of interest [12], which could significantly bias the stiffness measurement. The propagation mode was employed to further improve the data reliability by confirming shear wave propagation, as expected. During the stiffness measurement, a circle with a diameter of 1 cm was placed in the center of the region of interest (ROI), and the mean elasticity (Emean) inside the circle was considered for analysis (Figure 1).

**Figure 2.**
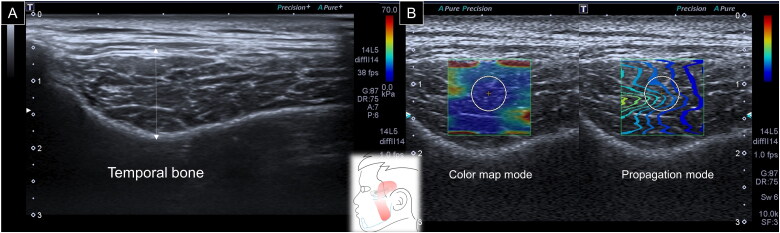
Ultrasound imaging for thickness **(A)** and stiffness **(B)** measurements of the temporalis muscle. White dashed line, maximal muscle thickness.

When evaluating the temporalis muscle, a linear transducer was first placed in the horizontal plane over the zygomatic arch. The transducer was relocated cranially until the deepest portion of the temporalis fossa was visible. The thickness of the temporalis muscle was defined as the distance between the superficial fascia of the temporalis and the periosteum [9]. Subsequently, the SWS mode was initiated, and a circle with a diameter of 1 cm was positioned in the middle of the ROI to acquire muscle stiffness (Figure 2).

**Figure 3.**
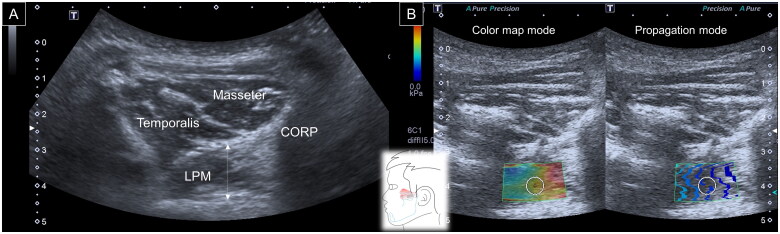
Ultrasound imaging for thickness **(A)** and stiffness **(B)** measurements of the temporalis muscle. White dashed line, maximal muscle thickness; LPM, lateral pterygoid muscle; CORP, coronoid process of the mandible.

For the lateral pterygoid muscle, a curved transducer was positioned in the infrazygomatic region. The participant was then asked to open the mouth to enlarge the window between the zygomatic arch and mandibular notch. The muscle was triangular in shape, emerging from the coronoid process of the mandible and attached to the lateral pterygoid plate. Thickness was defined as the distance between the superficial and deep muscle fasciae crossing the muscle midpoint. Similar to the former two stiffness measurements, a circle with a diameter of 1 cm was positioned in the middle of the ROI following a shift to the SWS mode (Figure 3).

### Reliability test

The reliability, expressed by the intra-class correlation coefficient (ICC) and analyzed by the two-way mixed model [14], was evaluated in the first 10 participants without orofacial pain. Regarding intra-rater reliability, the primary investigator conducted the thickness and stiffness measurements of all target muscles twice, with an interval of two hours between the two sessions. On the other hand, the second investigator performed the ultrasound assessment soon after the first examination session of the primary investigator and the data were employed for inter-rater reliability.

### Sample size estimation

The sample size for this study was determined using data from a previous study [15] that employed SWS to assess masticatory muscle stiffness both before and after conservative treatment. In individuals without orofacial pain, it was assumed that the mean tongue stiffness was 12 ± 3 kPa. It was also hypothesized that patients experiencing orofacial pain might show an average increase of 2 kPa in masseter elasticity. To achieve a statistical power of 80% and maintain a significance level of 0.05 (alpha value), a 1:1 subject ratio was established. Consequently, the study required the inclusion of 70 subjects in total.

### Statistical analysis

Continuous variables are denoted by mean and standard deviation and analyzed by Analysis of Variance (ANOVA) for normally distributed data or by Kruskal-Wallis test for non-normally distributed data. Data normality was tested using the Shapiro-Wilk test. Categorical variables are shown as proportions and were investigated using Chi-square or Fisher's exact tests (in case of sparse data). The generalized estimating equation (GEE) [16], suitable for the management of auto-correlated data, was employed to adjust for the influence of age, sex, laterality, and body mass index (BMI) on muscle thickness and stiffness. The focused covariates were the presence of orofacial pain and pain on the examined side. All analyses were performed using SPSS (version 21.0. Chicago, SPSS Inc.) software, and statistical significance was set at a *p* < 0.05.

## Results

A total of 98 subjects were included: 48 asymptomatic controls, 13 patients with unilateral pain, and 37 patients with bilateral orofacial pain. The three groups were similar in terms of age, BMI, and maximal mouth opening (Table 1).

**Table 1.** Characteristics of the enrolled participants.

	Asymptomatic Controls (*n* = 48)	Unilateral Pain (*n* = 13)	Bilateral Pain (*n* = 37)	*p* value
Female (%)	24 (50.00%)	9 (69.23%)	26 (70.27%)	0.129
Age (years)	51.00 ± 17.16 (46.02 to 55.98)	48.62 ± 15.09 (39.50 to 57.74)	49.30 ± 16.57 (43.77 to 54.82)	0.820
Body mass index (kg/m^2^)	23.11 ± 2.84 (22.29 to 23.94)	22.78 ± 2.29 (21.39 to 24.16)	23.06 ± 4.66 (21.50 to 24.61)	0.515
Maximum pain-free mouth opening (mm)	47.29 ± 5.38 (45.73 to 48.85)	42.69 ± 5.72 (39.24 to 46.15)	45.11 ± 8.52 (42.27 to 47.95)	0.069
Numeric rating scale (right facial region)	0.00 ± 0.00 (0.00 to 0.00)	1.15 ± 1.57 (0.20 to 2.10)	3.35 ± 2.00 (2.68 to 4.02)	<0.001*
Numeric rating scale (left facial region)	0.00 ± 0.00 (0.00 to 0.00)	1.38 ± 1.80 (0.29 to 2.48)	3.27 ± 1.97 (2.61 to 3.93)	<0.001*
Masseter area tenderness (n, right facial region)	0 (0.00%)	6 (46.15%)	37 (100.00%)	<0.001*
Masseter area tenderness (n, left facial region)	0 (0.00%)	7 (53.85%)	36 (97.30%)	<0.001*
Temporalis area tenderness (n, right facial region)	0 (0.00%)	0 (0.00%)	12 (32.43%)	<0.001*
Temporalis area tenderness (n, left facial region)	0 (0.00%)	2 (15.38%)	12 (32.43%)	<0.001*

*p < 0.05.

The values of continuous variables were expressed by the mean and standard deviation (95% confidence interval of mean). The values of categorical variables were expressed by the number (percentage).

The intra-rater reliability values for thickness measurements of the masseter, temporalis, and lateral pterygoid muscles were 0.976 (95% confidence interval [CI], 0.939–0.991), 0.897 (95% CI, 0.682–0.969), and 0.966 (95% CI, 0.893–0.990), respectively. The inter-rater reliability values for thickness measurements of the masseter, temporalis, and lateral pterygoid muscles were 0.957 (95% CI, 0.891–0.983), 0.724 (95% CI, 0.285–0.912), and 0.844 (95 CI, 0.566–0.950), respectively. The intra-rater reliability values for the stiffness measurements of the masseter, temporalis, and lateral pterygoid muscles were 0.893 (95% CI, 0.630–0.972), 0.862 (95% CI, 0.540–0.964), and 0.874 (95% CI, 0.573–0.967), respectively. The inter-rater reliability values for stiffness measurements of the masseter, temporalis, and lateral pterygoid muscles were 0.805 (95% CI, 0.393–0.948), 0.802 (95% CI, 0.387–0.947), and 0.745 (95% CI, 0.259–0.930), respectively.

In the group with unilateral orofacial pain, no significant differences were identified in the thickness and stiffness of the three examined muscles (painful vs. asymptomatic sides) (Table 2). Likewise, thickness and stiffness were not significantly different between the right and left sides in each of the three groups (Table 3).

**Table 2.** Muscle thickness/stiffness in patients with unilateral orofacial pain.

	Painful site	Non-painful site	*p* value
**Thickness**			
Masseter muscle (mm)	12.04 ± 1.31 (11.25 to 12.84)	11.87 ± 1.73 (10.83 to 12.92)	0.576
Temporalis muscle (mm)	5.35 ± 0.63 (4.97 to 5.73)	5.48 ± 0.82 (4.99 to 5.98)	0.442
Lateral pterygoid muscle (mm)	14.56 ± 1.23 (13.82 to 15.31)	14.62 ± 1.32 (13.83 to 15.42)	0.285
**Stiffness**			
Masseter muscle (kPa)	14.68 ± 5.30 (11.48 to 17.89)	11.21 ± 4.31 (8.60 to 13.81)	0.101
Temporalis muscle (kPa)	34.49 ± 15.77 (24.96 to 44.02)	26.17 ± 9.63 (20.35 to 31.99)	0.221
Lateral pterygoid muscle (kPa)	34.18 ± 9.89 (28.20 to 40.15)	31.96 ± 12.64 (24.32 to 39.60)	0.552

*p < 0.05.

The values of continuous variables were expressed by the mean and standard deviation (95% confidence interval of mean).

**Table 3.** Comparative muscle thickness/stiffness values of the subjects.

	Right side	Left side	*p* value
**Asymptomatic Controls (n = 48)**			
Thickness			
Masseter muscle (mm)	12.09 ± 2.10 (11.48 to 12.70)	12.05 ± 2.09 (11.45 to 12.66)	0.829
Temporalis muscle (mm)	5.72 ± 0.91 (5.46 to 5.99)	5.56 ± 0.68 (5.37 to 5.76)	0.261
Lateral pterygoid muscle (mm)	16.52 ± 1.17 (16.18 to 16.86)	16.24 ± 1.39 (15.84 to 16.65)	0.054
Stiffness			
Masseter muscle (kPa)	9.90 ± 2.56 (9.15 to 10.64)	9.24 ± 2.48 (8.52 to 9.95)	0.074
Temporalis muscle (kPa)	34.48 ± 19.19 (28.91 to 40.05)	40.52 ± 23.99 (33.55 to 47.49)	0.115
Lateral pterygoid muscle (kPa)	33.07 ± 14.15 (28.97 to 37.18)	32.93 ± 13.59 (28.99 to 36.88)	0.894
**Patients with Unilateral Oral Facial Pain (n = 13)**			
Thickness			
Masseter muscle (mm)	12.21 ± 1.48 (11.31 to 13.10)	11.71 ± 1.55 (10.77 to 12.64)	0.087
Temporalis muscle (mm)	5.42 ± 0.83 (4.92 to 5.92)	5.42 ± 0.63 (5.04 to 5.79)	0.944
Lateral pterygoid muscle (mm)	14.63 ± 1.32 (13.83 to 15.42)	14.56 ± 1.23 (13.81 to 15.30)	0.109
Stiffness			
Masseter muscle (kPa)	14.05 ± 5.62 (10.66 to 17.45)	11.84 ± 4.35 (9.21 to 14.47)	0.279
Temporalis muscle (kPa)	31.82 ± 16.55 (21.81 to 41.82)	28.85 ± 10.01 (22.80 to 34.89)	0.917
Lateral pterygoid muscle (kPa)	35.05 ± 9.88 (29.07 to 41.02)	31.09 ± 12.42 (23.59 to 38.60)	0.196
**Patients with Bilateral Oral Facial Pain (n = 37)**			
Thickness			
Masseter muscle (mm)	11.75 ± 1.74 (11.17 to 12.33)	11.49 ± 1.91 (10.85 to 12.13)	0.161
Temporalis muscle (mm)	5.36 ± 0.72 (5.12 to 5.60)	5.26 ± 0.88 (4.97 to 5.55)	0.377
Lateral pterygoid muscle (mm)	14.65 ± 1.38 (14.19 to 15.11)	14.34 ± 1.37 (13.88 to 14.79)	0.052
Stiffness			
Masseter muscle (kPa)	15.64 ± 7.50 (13.13 to 18.14)	14.46 ± 7.90 (11.83 to 17.10)	0.074
Temporalis muscle (kPa)	34.45 ± 13.16 (30.06 to 38.84)	40.29 ± 25.81 (31.68 to 48.89)	0.222
Lateral pterygoid muscle (kPa)	33.82 ± 12.12 (29.78 to 37.86)	34.97 ± 10.34 (31.52 to 38.42)	0.236

*p < 0.05.

The values of continuous variables were expressed by the mean and standard deviation (95% confidence interval of mean).

The GEE for analyzing the associations of muscle thickness and stiffness with demographics and orofacial pain is shown in Table 4. Female sex was associated with decreased thickness of the masseter (*p* = 0.002) and temporalis (*p* = 0.039) muscles. There was an inverse association between age and masseter (*p* = 0.026) and temporalis (*p* = 0.033) muscles. BMI was positively associated with masseter (*p* < 0.001) and temporalis (*p* < 0.001) muscle thickness and negatively associated with masseter (*p* = 0.015) and lateral pterygoid (*p* = 0.045) muscles. Muscles on the right side (in comparison with the left side) were associated with increased lateral pterygoid muscle thickness (*p* = 0.010) and masseter (*p* = 0.003) muscle stiffness and negatively associated with temporalis (*p* = 0.016) muscle stiffness.

**Table 4.** Associations between patient demographics and masticatory muscle thickness/stiffness values.

	Thickness			Stiffness		
	Masseter muscle	Temporalis muscle	Lateral pterygoid muscle	Masseter muscle	Temporalis muscle	Lateral pterygoid muscle
Female	−1.018(−1.659 to −0.377)	−0.277(−0.541 to −0.014)	−0.298(−0.802 to 0.206)	0.124(−1.912 to 2.16)	−4.949(−12.321 to 2.423)	−2.398(−6.95 to 2.153)
	***p* = 0.002** *	***p* = 0.039** *	*p* = 0.247	*p* = 0.905	*p* = 0.188	*p* = 0.302
Age	−0.021(−0.040 to −0.003)	−0.008(−0.016 to −0.001)	0.001(−0.014 to 0.015)	−0.009(−0.069 to 0.051)	−0.141(−0.358 to 0.077)	−0.011(−0.145 to 0.124)
	***p* = 0.026** *	***p* = 0.033** *	*p* = 0.942	*p* = 0.764	*p* = 0.204	*p* = 0.878
BMI	0.259(0.172 to 0.346)	0.081(0.045 to 0.117)	−0.018(−0.087 to 0.050)	0.344(0.067 to 0.621)	0.505(−0.498 to 1.508)	0.634(0.015 to 1.253)
	***p* < 0.001** *	***p* < 0.001** *	*p* = 0.603	***p* = 0.015** *	*p* = 0.324	***p* = 0.045** *
Right side	0.186(−0.015 to 0.387)	0.115(−0.025 to 0.255)	0.262(0.062 to 0.462)	1.097(0.384 to 1.811)	−4.677(−8.477 to −0.878)	0.181(−2.161 to 2.523)
	*p* = 0.070	*p* = 0.107	***p* = 0.010** *	***p* = 0.003** *	***p* = 0.016** *	*p* = 0.880
Presence of Oral Myofascial Pain	−0.267(−1.044 to 0.510)	−0.127(−0.523 to 0.269)	−1.751(−2.417 to −1.085)	1.804(−0.760 to 4.368)	−9.050(−19.970 to 1.870)	−0.372(−7.109 to 6.364)
	*p* = 0.501	*p* = 0.528	***p* < 0.001** *	*p* = 0.168	*p* = 0.104	*p* = 0.914
Side of Pain	0.114(−0.417 to 0.645)	−0.147(−0.493 to 0.199)	−0.057(−0.574 to 0.46)	3.603(1.736 to 5.471)	9.055(−0.399 to 18.509)	2.252(−3.577 to 8.081)
	*p* = 0.674	*p* = 0.404	*p* = 0.829	***p* < 0.001** *	*p* = 0.060	*p* = 0.449

*p < 0.05. Data are expressed by the beta coefficients and their 95% confidence intervals (analyzed by a generalized estimating equation).

BMI: body mass index.

**Figure 4.**
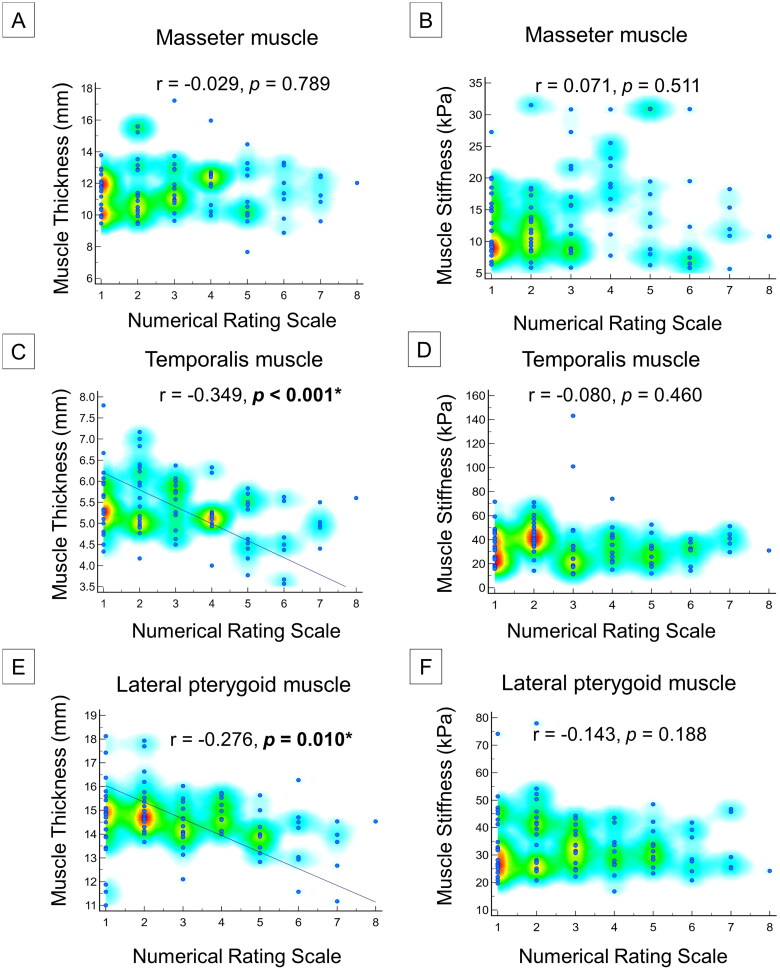
Correlations between muscle thickness/stiffness and numeric rating scale for masseter **(A, B)**, temporalis **(C, D)** and lateral pterygoid **(E, F)** muscles.

In addition, the presence of orofacial pain was associated with a decrease in the lateral pterygoid muscle thickness (*p* < 0.001). Orofacial pain was significantly associated with masseter muscle stiffness on the same side (*p* < 0.001). Increased temporalis muscle stiffness (*p* = 0.06) was also observed on the painful sides (Table 4). Numeric rating scale scores were negatively correlated with temporalis (*p* < 0.001) and lateral pterygoid (*p* = 0.001) muscle thicknesses, but not with any of the stiffness measurements (Figure 4).

## Discussion

Our study has yielded several noteworthy results. Firstly, we have established the reliability of SWS as a dependable tool for assessing masticatory muscle stiffness. Secondly, we observed a significant association between orofacial pain and heightened stiffness in the masseter muscle, with a parallel trend detected in the temporalis muscle. Thirdly, our analysis revealed no correlation between the intensity of pain and masticatory muscle stiffness.

Regarding the measurement of muscle stiffness, the ICC of intra-rater reliability ranged between 0.862 and 0.893, whereas that of inter-rater reliability ranged between 0.745 and 0.805. Of note, an ICC of 0.75–0.9 is considered as good reliability [14]. Our study demonstrated that SWS is a reliable imaging tool to noninvasively assess the elastic properties of masticatory muscles. Similar to SWS, which quantifies tissue stiffness based on the shear wave velocity perpendicular to the acoustic impulse, another commonly used mode is strain sonoelastography [17,18]. However, it has a disadvantage of significant dependence on tissue deformation during manual compression, which can vary indisputably across different examiners. Furthermore, manual compression is usually applied in a gentle and homogenous manner, which is difficult to transmit to deeper structures, such as the lateral pterygoid muscle. Our observation was also consistent with an antecedent ultrasound study, which reported that the intra-observer agreement concerning shear wave measurements of the masseter muscle could reach 0.93 with trained radiologists [19].

After adjusting for age, sex, and laterality, our findings showed that orofacial pain was associated with increased stiffness of certain masticatory muscles. A recent sonoelastography study compared 19 patients with neck pain and myofascial trigger points in the trapezius muscles with 34 asymptomatic participants [20]. Increased muscle stiffness was observed at the target points of the patients compared with the reference group. Another study investigated 30 women with trigger points in the upper trapezius and revealed a significant decrease in SWS-measured muscle stiffness following therapeutic exercise [21]. Both aforementioned studies indicated that muscles tended to be stiffer in the presence of myofascial pain when compared with the non-painful status. In muscles with myofascial pain, taut bands, that is, contracture of sarcomere units and decreased regional blood flow, are common. Lok et al. employed probe oscillation SWS and reported that muscles with taut bands had higher shear wave velocities than those without [22]. The authors speculated that the increase in stiffness was due to persistent local muscle contraction.

Among the three muscles examined, the masseter muscle was found to be the most relevant to increased stiffness in patients with orofacial pain. This quadrangular muscle, emerging from the zygomatic arch and inserting into the mandibular ramus, offers forceful mandibular elevation and protrusion during chewing. In adolescents with moderate/severe temporo-mandibular joint disorders, electromyographic activity of the masseter muscle was found to be increased [23]. Likewise, another surface electromyography study identified that the mean resting masseter activity was capable of discriminating children with and without pain-related temporo-mandibular joint disorder, with an area under the curve of 0.662 [24]. Considering the close association between temporo-mandibular joint disorders and orofacial pain, it appears plausible that the masseter muscle was the most involved muscle in our patient group, together with increased stiffness on sonoelastography.

A trend of increased stiffness was observed in the temporalis muscle on the painful side. It has a broad fan-like shape emerging from the temporalis fossa and converging on the coronoid process of the mandible. Its contraction results in elevation and retrusion of the mandible. Bruxism has been reported to be associated with pain/hypertrophy of the temporalis muscle, which can be alleviated by botulinum toxin injections [25]. An antecedent systematic review pointed out that the threshold of pressure pain in the temporalis was higher than that in the masseter, indicating that the former is less involved in orofacial pain [26]. Interestingly, there is a gradient of pressure pain sensitivity rising from the posterior (in the temporalis fossa) to the anterior aspect (near the zygomatic arch) [27]. Therefore, our sonoelastography findings could be explained by the possibly lower prevalence of associated temporalis pain (than the masseter) and heterogeneous spatial distribution of pain thresholds.

Although orofacial pain was associated with decreased lateral pterygoid muscle thickness, no association was identified with respect to stiffness. The lateral pterygoid muscle has two heads, emerging from the infratemporal crest of the greater wing and the lateral surface of the lateral pterygoid plate of the sphenoid bone attaching to the temporo-mandibular joint capsule and pterygoid fovea on the condyloid process of the mandible, respectively [28]. Its contraction contributes to the mandibular protrusion and depression. A magnetic resonance imaging study revealed increased signal intensity at the superior head in patients with temporo-mandibular joint disc displacement [29]. An ultrasound study showed the reliability of lateral pterygoid muscle thickness measurements [9], which could be challenging in painful muscles with disuse atrophy. Moreover, unlike the masseter and temporalis muscles, the lateral pterygoid is situated deeper and runs an oblique course in relation to the skin. Its muscle fiber direction was shown to have a major influence on the shear wave velocity [30]. As such, standardization of the angle between the transducer footprint and the muscle fibers is difficult and can make SWS unable to distinguish between patients with and without orofacial pain.

Our study also identified female participants as having a decreased masseter thickness. This finding was consistent with an antecedent ultrasound study [9], which showed thicker masticatory muscles in males than females. The possible mechanism could be sex-related differences in facial length, that is, higher in men than in women [31]. Furthermore, age was associated with reduced masseter and temporalis muscle thicknesses. The aging population is vulnerable to decreased muscle mass/function, namely sarcopenia, which can be observed in both appendicular and swallowing-related muscles [32]. Our finding might be explained by a previous computed tomography study that reported high correlations between skeletal muscle mass indices of the masticatory and abdominal/paraspinal muscles [33]. Interestingly, we also found that BMI was positively associated with masseter and temporalis muscle thickness and negatively associated with masseter and lateral pterygoid muscle stiffness. As a higher BMI indicates a larger body size, it appears reasonable that BMI was positively correlated with muscle thickness. Regarding the relationship between BMI and muscle stiffness, a recent study employed a MyotonPRO device to assess biceps brachii and biceps femoris muscles and reported a positive correlation between BMI and muscle tonicity [34]. A possible explanation is that an increased BMI leads to escalated loading stimuli, further causing higher muscle stiffness.

In addition, laterality was likely to affect lateral pterygoid muscle thickness and masseter/temporalis muscle stiffness. Asymmetry in lateral preference for mastication was revealed in an electromyographic study [35]. Although the majority of previous ultrasound studies demonstrated a limited role of laterality in influencing the thickness of the masseter muscles [36], they were conducted in asymptomatic volunteers. In our study, the variation and location of orofacial pain may have complicated muscle laterality. Accordingly, examiners should be cautious when interpreting the results, especially when comparing different sides. Finally, although the numeric rating scale was negatively associated with certain masticatory muscle thickness values, it did not correlate with any muscle stiffness values. This finding is in line with a recent ultrasound study that showed thinner masseter muscles in patients with temporo-mandibular joint disorders (in asymptomatic controls) [37]. The aforementioned study also found differences in the masseter muscle tone between the patient and control groups. Likewise, considering the relationship between such disorders and orofacial pain, muscle stiffness measurements could have been affected in our study, eventually not correlating with pain intensity.

To our knowledge, our study is one of the first to use SWS to investigate the relationship between orofacial pain and the mechanical properties of the masseter. We conducted a thorough evaluation involving 98 participants, including controls and those with unilateral or bilateral orofacial pain, establishing the reliability of SWS to quantify muscle stiffness. There is evidence of a statistically significant association between orofacial pain and increased stiffness in the masseter muscle. It is therefore possible to objectively and quantitatively assess orofacial pain, with SWS as a tool for evaluating treatment outcomes in this field.

This study has several limitations that need to be acknowledged. First, although the medial pterygoid muscle belongs to the masticatory group, the echotexture and stiffness were not assessed. The main reason was its anatomic location, being deep in the pterygoid plate and making it invisible through the infra-zygomatic window. Second, the intensity of orofacial pain was quantified using a subjective numeric rating scale reported by the participants. In the future, the use of a pressure gauge to map the pain threshold would better represent the status of the individual muscles. Third, changes in muscle stiffness following intervention (such as dry needling) were not assessed in this cross-sectional study. As such, further research should incorporate longitudinal and postprocedural follow-ups to examine relevant/temporal changes.

## Conclusions

SWS reliably assesses masticatory muscle stiffness. The side affected by orofacial pain, notably the ipsilateral masseter muscles, exhibited heightened stiffness, with a similar trend observed in the temporalis muscle. Interestingly, lateral pterygoid muscle stiffness appeared to be largely unaffected. These findings pave the way for future research utilizing our sonoelastography protocol to investigate muscle changes after interventions, offering promising prospects for improving orofacial pain management.

## Data Availability

The datasets generated or analyzed during the study are available from the corresponding author on reasonable request.

## Abbreviations

IASP: International Association for the Study of Pain
SWS: sonoelastography
ROI: region of interest
Emean: mean elasticity
ICC: intra-class correlation coefficient
ANOVA: analyzed by Analysis of Variance
GEE: generalized estimating equation
BMI: body mass index
CI: confidence interval.
